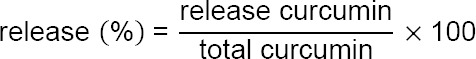# Curcumin-Loaded PLA Nanoparticles: Formulation and Physical Evaluation

**DOI:** 10.3797/scipharm.ISP.2015.10

**Published:** 2016-02-14

**Authors:** Heni Rachmawati, Yulia L. Yanda, Annisa Rahma, Nobuyuki Mase

**Affiliations:** 1 School of Pharmacy, Bandung Institute of Technology, Ganesha 10, 40322, Bandung, Indonesia; 2 Department of Applied Chemistry and Biochemical Engineering, Graduate school of Engineering, and Green Energy Research Division, Research Institute of Green Science and Technology, Shizuoka University 3-5-1 Johoku, Hamamatsu, Shizuoka 432-8561, Japan

**Keywords:** Curcumin, poly(lactic acid), PLA, TPGS, Nanoparticle formulation

## Abstract

Curcumin is a polyphenolic compound derived from *Curcuma domestica* (Zingiberaceae) that possesses diverse pharmacological effects including anti-inflammatory, antioxidant, antimicrobial, and anticarcinogenic activities. Although phase I clinical trials have shown curcumin as a safe drug even at high doses (12 g/day) in humans, poor bioavaibility largely limits its pharmacological activity. Nanoencapsulation in biodegradable polymers is a promising alternative to improve curcumin bioavaibility. In this study, curcumin was encapsulated in biodegradable polymer poly-(lactic acid) (PLA) nanoparticles via the emulsification-solvent evaporation method. Optimization of selected parameters of this method including the type of solvent, surfactant concentration, drug loading, sonication time, and centrifugation speed, were performed to obtain polymeric nano-carriers with optimum characteristics. Dichloromethane was used as the solvent and vitamin E polyethylene glycol succinate (TPGS) was used as the surfactant. Four minutes of sonication time and centrifugation at 10500 rpm were able to produce spherical nanoparticles with average size below 300 nm. The highest encapsulation efficiency was found on PLA nanoparticles containing 5% of curcumin at 89.42 ± 1.04%. The particle size, polydispersity index, zeta potential of 5% curcumin-PLA nanoparticles were 387.50 ± 58.60 nm, 0.289 ± 0.047, and −1.12 mV, respectively. Differential Scanning Calorimetry (DSC) and X-Ray Diffraction (XRD) studies showed partial interaction between the drug and polymer.

## Introduction

Curcumin is a polyphenolic compound derived from the rhizome of *Curcuma domestica* (Zingiberaceae). Curcumin has a wide spectrum of biological and pharmacological effects including anti-inflammation, antioxidant, antimicrobe, and anticarcinogen. Phase I clinical trials have shown curcumin as a safe drug even at high doses (12 g/day) in human. However, lack of aqueous solubility, poor tissue absorption, rapid metabolism and quick systemic removal lead to low bioavaibility of curcumin. Hence, the development of a delivery system which can facilitate the administration of curcumin in an aqueous medium will significantly improve the clinical efficacy of curcumin [1].

**Fig. 1:**
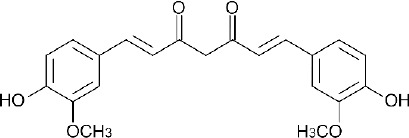
Chemical structure of curcumin

Polymeric nanoparticles have attracted significant attention in the study of drug delivery systems as they offer a means for localized or targeted delivery systems of a drug to a specific tissue/organ sites of interest with an optimal release rate [2]. Poly (lactide acid) (PLA) is FDA (US Food and Drug Administration) – approved biodegradable polymer, which is most often used in studies of drug delivery due to their very low toxicity. D-α-tocopheryl polyethylene glycol 1000 succinate (TPGS), derived from natural vitamin E (α-tocopheryl), is a safe and effective form of vitamin E for reversing or preventing vitamin E deficiency due to its high oral bioavailability. It has many other potential applications, such as solubilizer, absorption enhancer and vehicle for lipid-based drug delivery formulations. TPGS is readily absorbed in the gastrointestinal tracts, and inhibits P-glycoprotein, the multidrug transporter in the intestine to enhance the cytotoxicity of curcumin. Significant potential use has been identified for TPGS in the application of nanoparticle and lipid-based formulation. The chemical structure of vitamin E TPGS comprises both lipophilicity and hydrophilicity, resulting in amphiphilic properties. Its lipophilic alkyl tail and hydrophilic polar head portion are bulky and have a large surface area. Such characteristics makes TPGS a good emulsifier which is able to emulsify a wide range of water-oil immiscible systems [3, 4].

In this study, we formulated curcumin in poly (Lactic acid) (PLA)-based nanocarrier using emulsification-solvent evaporation method. Several processing parameters were investigated including type of solvent, surfactant concentration, sonication time, centrifugation speed, and amount of drug in order to obtain curcumin-PLA nanoparticles with satisfying properties. The ability of PLA to encapsulate curcumin was also investigated by using FTIR spectroscopy, which will inform whether chemical interaction between PLA and curcumin molecules occurred. The final aim of this study is to improve curcumin bioavailability and stability, thereby increasing the therapeutic efficacy.

## Results and Discussion

### Type of Solvent

The formation of an emulsion is the most important step in nanoparticle preparation because the size of emulsion droplet is directly related to the nanoparticle size. This emulsion, which is formed by mixing an organic phase consisting of polymer with an aqueous phase containing a surfactant or stabilizer, is broken down into droplets by applying external energy, and these nanodroplets lead to nanoparticle formation upon evaporation of the organic solvent [5].

The most important parameters of solvent in organic phase are polarity and boiling point. These factors influence the process of nanoparticle formation. The most commonly used solvents for PLA are ethyl acetate and dichloromethane. Both solvents are easily removed and suitable to dissolve polymer. Ethyl acetate is relatively less toxic than dichloromethane, but dichloromethane produced smaller particle with narrow size distribution over ethyl acetate (Table 1).

**Tab. 1:**
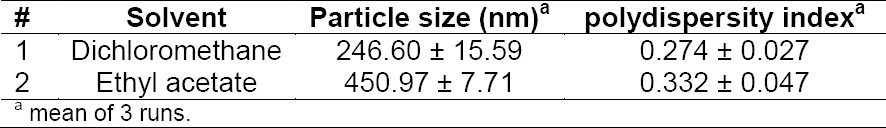
Effect of solvent on particle size and polydispersity index

### Sonication time

Table 2 shows the influence of sonication time on the size of nanoparticles. Four minutes sonication resulted in the smallest diameter. The duration of sonication correlated with the energy for emulsification process. The longer the sonication, the higher the emulsification energy leading to particle aggregation hence increasing the particle size and polydispersity index.

**Tab. 2:**
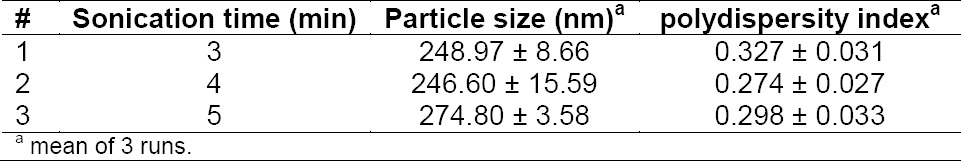
Effect of sonication time on particle size and polydispersity index

### Surfactant Concentration

The amount of surfactant plays an important role in the emulsification – solvent evaporation process for the protection of droplets against coalescence [6]. SEM analysis showed the formation of a stable layer on the surface of the nanoparticles which cannot be removed during washing procedure, indicating that vitamin E TPGS as the surfactant sufficiently protected the particles from aggregation.

**Fig. 2:**
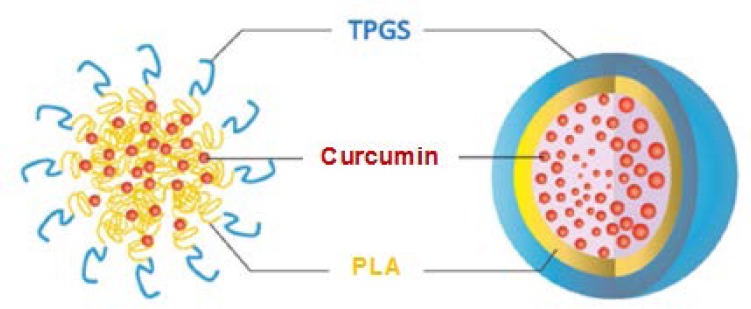
Schematic illustration of curcumin-loaded PLA nanoparticles

An increase in vitamin E TPGS concentration up to 0.03% caused a significant decrease in nanoparticle size. A reduced interfacial tension was thought to result in decreased particle size. At concentration above 0.03%, the particle size increased due to viscosity enhancement of aqueous phase. This results in a decreased net shear stress and subsequently increased the particle size.

**Tab. 3:**
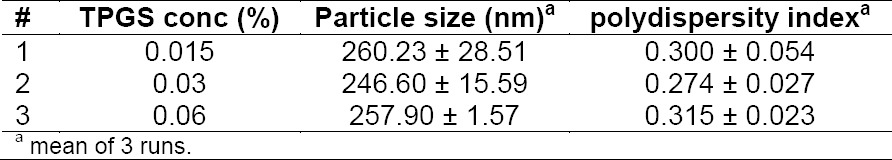
Effect of surfactant concentration on particle size and polydispersity index

### Centrifugation speed

Centrifugation speed influenced the yield amount of nanoparticles. The faster the centrifugation speed, the smaller size of nanoparticles can be obtained. At 6000 to 10500 rpm of centrifugation speed, the nanoparticles had a spherical shape with no aggregation. However, aggregation occurred at 12000 rpm. Too high centrifugation speed cause strongly aggregated particles in the form of a pellet that difficult to redisperse.

**Tab. 4:**
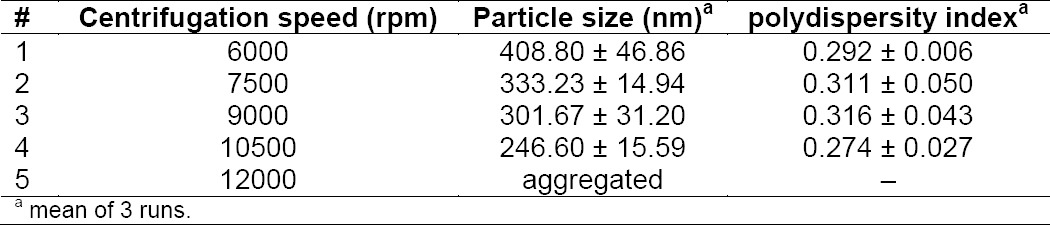
Effect of centrifugation speed on particle size and polydispersity index

### Drug Amount

Table 5 summarizes the effect of curcumin amount on nanoparticle characteristics (particle size, polydispersity index, zeta potential, and encapsulation efficiency). It is clear that the more drug loaded to polymeric matrix, the larger the size of nanoparticles obtained. Meanwhile, the effect of drug amount on encapsulation efficiency did not follow a linear trend. As the amount of drug increased from 0 to 10%, the percentage of encapsulated drug first increased. This means that more drug molecules could interact with the PLA molecules, resulting in an increase in the amount of curcumin encapsulated. However, the encapsulation efficiency continuously decreased when the amount of drug was more than 10%.

**Tab. 5:**
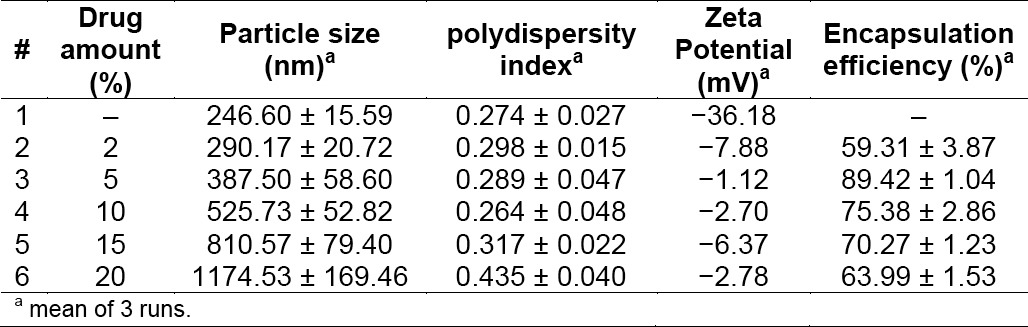
Effect of curcumin amount on nanoparticle characteristics

The ability of PLA to encapsulate curcumin might be due to the interaction between drug molecules and the PLA molecules. The surface of PLA nanoparticles has a negative charge and thus it is most likely to favor the electrostatic adsorption of polycations [7]. The negative surface charge of PLA nanoparticles originates from free carboxylic acid groups at the end of the PLA polymer chain. The presence of curcumin in PLA nanoparticles always reduced the negative zeta potential value. This is probably because a masking effect of the superficial carboxylic groups by the drug adsorbed on nanoparticle surface [8].

**Fig. 3:**
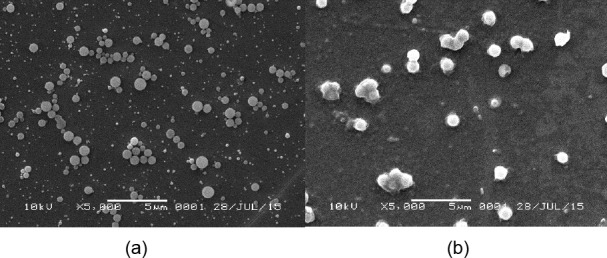
Scanning electron microscopic images of (a) blank PLA nanoparticles (5000x) (b) curcumin-loaded PLA nanoparticles (5000x)

### Particle Morphology by Scanning Electron Microscopy

The scanning electron microscopic images of the nanoparticles prepared by the emulsification-solvent evaporation method were found to be spherical in shape.

### Fourier Transform Infrared Spectroscopy

**Fig. 4:**
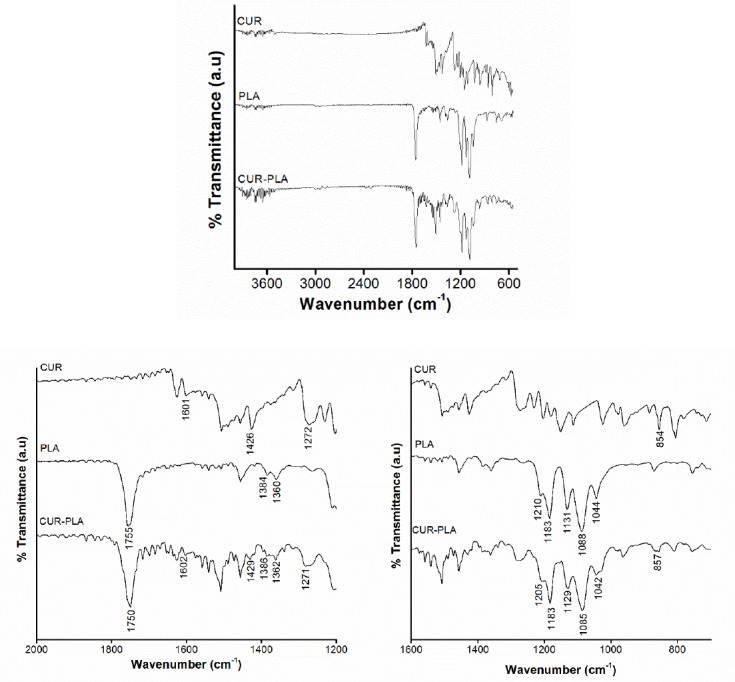
Fourier Transform Infrared spectroscopy for curcumin, blank PLA nanoparticles and curcumin-loaded PLA nanoparticles

FTIR spectra of curcumin, blank PLA nanoparticles, and curcumin-loaded PLA nanoparticles of optimized formulation are shown in Fig. 4. FTIR spectra of curcumin showed characteristic peaks at 3501 cm^−1^ corresponding to phenolic O-H stretching. However, this band cannot be distinguished with other peaks in curcumin-loaded PLA nanoparticles. Additionally, sharp peaks appeared at 1601 and 1427 cm^−1^ due to the stretching vibration of C=C of benzene ring and olefinic bending vibration of C-H bound to the benzene ring of curcumin. The shift of these peaks were observed from 1601 cm^−1^ to 1603 cm^−1^ and from 1426 cm^−1^ to 1429 cm^−1^, respectively. Moreover, the peak at 1272 cm^−1^ and 854 cm^−1^, assigned for vibration of C-O in –C-OCH_3_ of phenyl ring, was shifted to 1271 cm^−1^ and 857 cm^−1^, respectively in curcumin-loaded PLA. Some characteristics peaks of PLA and their shifts were identified. A needle like peak at 1755 cm^−1^ is assigned as carbonyl stretching C=O in the –CO-O- group of PLA and was observed at 1750 cm^−1^ at curcumin- loaded PLA nanoparticles. This shift indicated weak hydrogen bond formation between carbonyl group of PLA and hydroxyl group of curcumin. This finding is supported by the peak shift of the symmetric CH_3_ bending of PLA in curcumin-PLA nanoparticle, from at 1384 cm^−1^ to 1386 cm^−1^. C-H groups that showed symmetric CH bending were the neighboring groups to C=O in PLA. The changes in vibrational frequency indicated by the peak shift is probably the consequence of interaction between C=O group of PLA with O-H group of curcumin. A mountainous triplet peak at 1131, 1088, and 1044 cm^−1^, corresponding to C-O vibration in –CO-O- group in polymer chains, shifted accordingly to 1129, 1085, and 1042 cm^−1^, respectively. The peak shifts confirm that curcumin and PLA were bound together.

### Differential Scanning Calorimetry

**Fig. 5:**
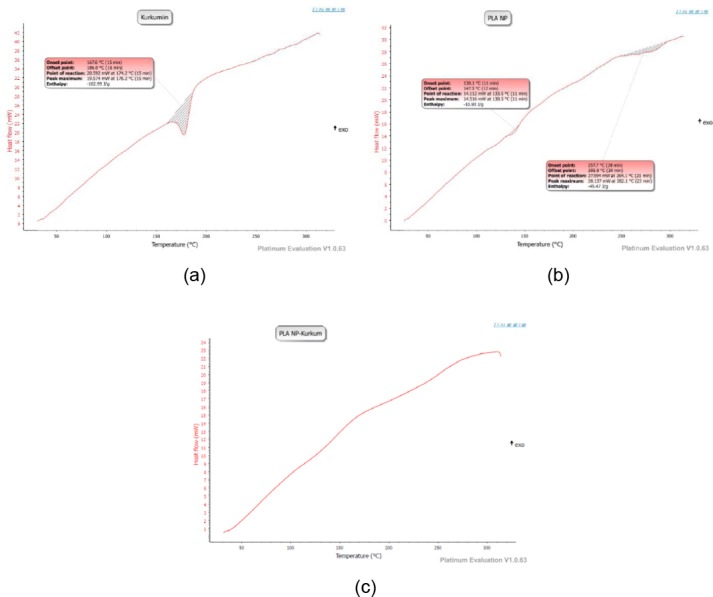
DSC profile of (a) curcumin, (b) blank PLA nanoparticles, (c) curcumin-loaded PLA nanoparticles

DSC thermogram of curcumin, blank PLA nanoparticles and curcumin-loaded PLA nanoparticles are shown in Figure 5. Thermogram of curcumin showed a sharp endothermic peak at 178.2°C which corresponds to the melting point of the drug, as expected. However, no melting peak was detected in curcumin-loaded PLA nanoparticles. This indicated that curcumin in the nanoparticles was in an amorphous or disordered crystalline phase or in the solid solution state, suggesting that the interaction between the drug and polymer occurs which is due to changes in physical form of crystalline to amorphous state.

### X-Ray Diffractometry

**Fig. 6:**
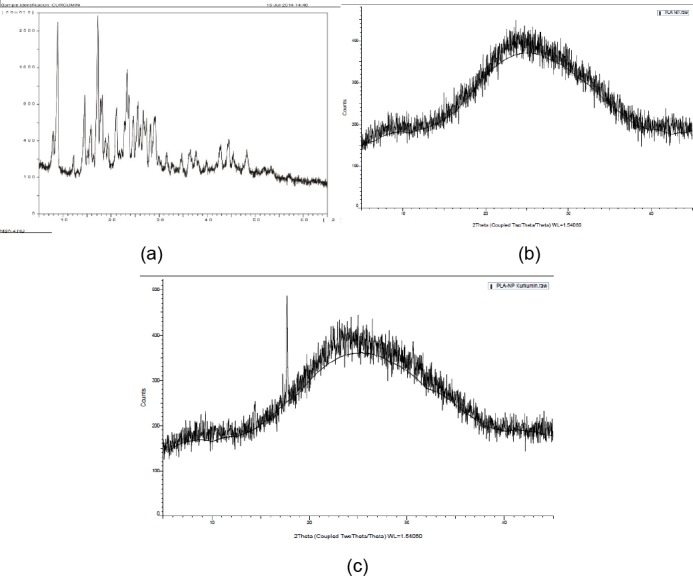
XRD pattern for (a) curcumin, (b) blank PLA nanoparticles, (c) curcumin-loaded PLA nanoparticles

To support the findings in DSC study, analysis of XRD patterns of curcumin, blank PLA nanoparticles, and curcumin-loaded PLA nanoparticles were performed (figure 6). The diffractogram of curcumin showed sharp and intense peaks of crystallinity. On the other hand, diffractogram of curcumin-loaded PLA nanoparticles showed reduction in number of peak intensity as compared to the curcumin, indicating decreased crystallinity or changes into amorphous phase of the drug. The disappearance of curcumin peaks confirms the successful loading of curcumin into the nanoparticles.

### In vitro Drug Release

**Fig. 7:**
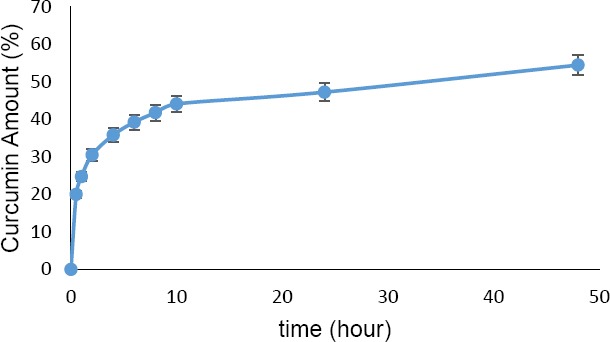
Drug release profile of curcumin-loaded PLA nanoparticles

The release profile of curcumin-loaded PLA nanoparticles in phosphate buffer saline (PBS) pH 7.4 at 37°C is shown in Fig. 7. The amount of drug released was determined for 48 hours. Initially, high release rate was observed, most likely resulting from drug molecules deposited on the surface of nanoparticles. Later, curcumin showed a relatively slower release rate after 10 hours. From the drug release profile, it is clear that 44.08% of curcumin was released from nanoparticles in the first 10 hours. After 24 hours, the amount of curcumin released was 47.21% and finally reached 54.43% in 48 hours.

## Experimental

### Materials

Curcumin was purchased from PT. Phytocemindo Lestari, Indonesia. Poly-(L-Lactic acid) (Mn:6400. Mw: 10700, PDI:1.67, enantiopurity : 87% ee) was obtained as a gift sample from Professor Nobuyuki Mase, Shizuoka University, Japan. Vitamin E TPGS, dichloromethane and methanol were purchased from Merck specialties Pvt. Ltd, Germany. Distilled water was purchased from PT. Brataco, Indonesia. Deionized water was purchased from Laboratory of Organic Chemistry, Bandung Institute of Technology, Indonesia. All other chemicals and reagents were of analytical grade and used as provided.

### Methods

#### Synthesis of PLA

PLA was synthesized by polymerizing a ring-opening polymerizable monomer in a compressive fluid with a metal-free organic catalyst to product a polymer. A microtube was charged with L-lactide, 4-dimethylaminopyridine (DMAP), and anhydrous ethanol. The micro tube was placed in a pressure-resistant container and heated to 60°C. Then, supercritical carbon dioxide (60°C, 10 MPa) was charged, followed by reaction at 60°C for 2 hours. Next, the pressure pump and the back pressure valve were used to adjust the flow rate at the outlet of the back pressure valve to 5.0 L/min. Then supercritical carbon dioxide was allowed to flow for 30 min. After the organic catalyst and the residual monomers had been removed, the reaction system was gradually returned to normal temperature and normal pressure. Three hours after, polymers particles in the container were taken out (Nemoto *et al.*, 2011).

#### Preparation of nanoparticles

Nanoparticles containing curcumin were prepared by emulsification – solvent evaporation method. Accurately weighed amount of curcumin and PLA were separately dissolved in DCM, then mixed. The organic solution was added into aqueous phase containing surfactant vitamin E TPGS using sonicator. After emulsification, the oil-in-water emulsion was stirred for 24 hours to evaporate the organic solvent. The nanoparticles formed were isolated by centrifugation for 15 minutes at 10500 rpm. Finally, the nanoparticles were washed with deionized water to remove the residual surfactant. The effects of various parameters on hydrodynamic particle size and percentage drug encapsulation efficiency were studied such as type of organic solvent, surfactant concentration in the aqueous phase, sonication time, centrifugation speed, and initial drug loading. Unless otherwise mentioned, all experiments were conducted by varying one parameter while keeping other parameters constant.

#### Characterization of Nanoparticles

Particle size and polydispersity index were determined in deionized water as a dispersion medium by photon correlation spectroscopy (Delsa™ Nano C Particle Analyzer, Beckman Coulter). Zeta potential was determined by electrophoretic light scattering.

#### Nanoparticle morphology

The nanoparticle surface appearance and shape of blank nanoparticles and curcumin-PLA nanoparticles were analyzed by scanning electron microscopy (SEM). Samples were prepared by finely spreading concentrated nanoparticle dispersion over object glass and by drying them overnight at room temperature. The samples were then coated in a cathodic evaporator with a fine gold layer and observed by SEM.

#### Differential Scanning Calorimetry (DSC)

Thermal behavior of curcumin, blank nanoparticles, and curcumin-PLA nanoparticles were determined by Differential Scanning Calorimetry. Accurately weighed samples were sealed in an aluminium pan and scanned at a temperature range of 30–300°C at the rate of 10°C/min.

#### X-ray diffraction (XRD)

X-ray diffraction analysis of curcumin, blank nanoparticles, and curcumin-PLA nanoparticles were carried out to characterize the physical form of curcumin in sample of optimized batch in an x-ray diffractometer (Bruker, 8D advance) with Cu Kα radiation. The diffraction angle 2θ was 5–45°.

#### FTIR Spectroscopy

Drug polymer interactions were studied by using Attenuated Total Reflectance IR spectrophotometer (Bruker α-E). FTIR analysis of pure drug, blank nanoparticles, and curcumin-loaded PLA nanoparticles were carried out without pre-treatment of samples. The spectrum was scanned from 4000–400 cm^−1^.

#### Encapsulation efficiency

The amount of curcumin encapsulated into nanoparticle was determined by UV-Vis Spectrophotometer (Beckman Du 7500i) at 424 nm. One milliliter of nanoparticle dispersion was centrifuged and the sediment was dried in a desiccator overnight. The dried sample was diluted in 1 mL of dichloromethane and the polymer was precipitated by adding 1 mL methanol. The solution was centrifuged for 30 minutes at 10500 rpm. One milliliter of supernatant then diluted in dichloromethane:methanol 1:1 (v/v) to adjust the absorbance. The procedure was also carried out for blank nanoparticles and the resulting solution was used as blank in UV-Vis analysis.

#### In vitro drug release

*In vitro* curcumin release from curcumin-loaded PLA nanoparticles were determined at pH 7.4. The nanoparticles were redispersed in phosphate-buffered saline solution (pH 7.4). Total volume was divided into 30 microcentrifuge tubes at 37°C under orbital shaking. At proper time intervals, curcumin in nanoparticles were centrifuged at 3000 rpm for 10 minutes and the sediment was extracted in methanol and quantified spectrophotometrically. Release of curcumin was quantified as follows: